# Impact of the COVID-19 pandemic crisis on turnover intention among nurses in emergency departments in Thailand: a cross sectional study

**DOI:** 10.1186/s12912-023-01495-3

**Published:** 2023-09-27

**Authors:** Songwut Sungbun, Siripan Naknoi, Panjasilpa Somboon, Orapan Thosingha

**Affiliations:** 1 Department of Adult and Gerontology Nursing, Princess Agrarajakumari College of Nursing, Chulabhorn Royal Academy, Bangkok, Thailand; 2 Department of Emergency Medical Service, Rajavithi Hospital, Bangkok, Thailand

**Keywords:** Burnout, Emergency nurses, Maladaptive regulation, Organization resources, Turnover intention

## Abstract

**Background:**

During the COVID-19 pandemic in Thailand, a large volume of COVID-19 patients were referred to hospital emergency departments (EDs). This increased job demand and job strain among ED nurses, resulting in a high risk of intention to leave their organization.

**Aims:**

To investigate turnover intention during the COVID-19 pandemic among ED nurses and examine the effect of organizational resources, maladaptive regulation, and job burnout on nurses’ turnover intention.

**Method:**

This cross-sectional study investigated 322 ED nurses. We divided participants into two groups: dark-red zone areas (pandemic crisis areas) and non-red zone areas (non-pandemic crisis areas). Descriptive statistics, Pearson’s correlation analysis, and multiple linear regression (forward stepwise method) were used to analyze factors that predicted turnover intention.

**Results:**

Most participants were female and the mean age was 34.54 years. During COVID-19 pandemic crisis, 72.8% of ED nurses in dark-red zone areas desired to leave their organization. The factors of motivation, exhaustion, and cognitive impairment positively influenced turnover intention among ED nurses in dark-red zone areas. Low availability of organizational resources was associated with an increase in the turnover intention rate. Maladaptive regulation, exhaustion, and cognitive impairment positively influenced turnover intention among ED nurses in non-red zone areas.

**Conclusion:**

Exhaustion and cognitive impairment stand as significant facets of burnout linked to the intention of turnover among ED nurses. To address this, we recommend fostering secure workplace settings and implementing interventions that alleviate job demands and strains for ED nurses, potentially reducing turnover intentions.

## Background

COVID-19 rapidly became widespread globally in 2021. In Thailand, the number of patients infected with COVID-19 quickly increased in the fourth wave and peaked in the middle of August 2021. To respond to the crisis, the Thai Government established the Center for COVID-19 Situation Administration (CCSA) that was the main diver of coordination, response, and implementation national COVID-19. The CCSA declared 29 “dark-red zone” provinces that were COVID-19 pandemic crisis areas [1]. However, the number of patients in hospitals was overwhelming, which directly impacted the healthcare system. As of September 2020, there were 126,313 patients admitted to hospital with COVID-19, of which 4,691 patients were in intensive care units. Moreover, vaccine supply limitations posed serious problems for distributing the vaccine to the general Thai population. At that time, 20.33% of Thai people had received the first dose of the COVID-19 vaccine and 5.59% had received the second dose [2].

Base on the job demands-resources (JD-R) theory and self-regulation model indicated that the effect of combination between high job demands and low job resources creates a highly stressful work environment led to enduring job burnout. The organizational resource and self-regulation may reduce and prevent job strain and burnout [3]. On the other hand, employees with high job demand and strain levels may be affected by maladaptive regulation of cognition and behaviors, including inflexible coping and self-undermining. When employees face elevated levels of job strain, they encounter challenges in maintaining concentration and are prone to making more work-related errors [3]. Recently, the theoretical model and result of previous studies had been explaining the role of job demand, organizational resource, self-regulation (emotional intelligence) and maladaptive regulation on burnout among nurses during COVID-19 pandemic [4–7]. Organizational support plays a critical role in the success of an organization, as it involves providing individuals with the necessary resources, reinforcement, encouragement, and effective communication to perform their roles effectively [7]. Nevertheless, the impact of organizational support on nurses’ intension to turnover during the Covid-19 pandemic need to be illustrated, the previous studies reported that a major adverse effect of long-term burnout among nurses was a significant association with high turnover intention [8–10].

The emergency department (ED) acts as the frontline response to any public health emergency. Nurses are the largest cadre of healthcare providers in the ED and must perform their day-to-day roles as well as managing situations of overcrowding [11]. In Thailand, the shortage of nurses is an attrition crisis, as indicated by a nurse to population ratio of 1:400, and the prediction of loss rate is approximately 7,000 nurses per year. Consequently, the projected figure indicates that the number of nurses in Thailand will decrease to 194,260 by 2026 [12]. The previous study revealed that high job demands increased intention to turnover among nurses during COVID-19 pandemic [10]. This high job demand coupled with low organizational resources, such as the lack of personal protective equipment (PPE) and unsustainable healthcare policies has been associated with exhaustion [4, 13]. Healthcare providers who experience exhaustion are still required to maintain physical, emotional, and cognitive effort while at work. Exhaustion among nurses have been related to an increased prevalence of mental health disorders and negative outcomes related to job performance [9, 10]. A systematic review demonstrated that frontline nurses are at a higher risk for mental disturbances compared to other types of healthcare providers [14]. As nurses provide direct care and are in frequent contact with patients, their increased exposure to contagion in the workplace may contribute to psychological challenges. [14–16]. Emergency nurses are additionally exposed to an elevated likelihood of emotional stress, unfavorable work conditions, and concern about infection due to their direct care for patients prior to diagnosis. The COVID-19 pandemic influenced organization resources as well as self-regulation of frontline nurses (e.g., inflexible coping and self-undermining) [7].

Since the onset of COVID-19 in November 2019, many studies have identified factors that influenced turnover intention among frontline nurses [8–10, 12], although no studies have compared the impact of the disruption in emergency care delivery due to COVID-19 between non-red zone areas (areas with low COVID-19 cases reported) and dark-red zone areas (COVID-19 pandemic crisis areas). During the COVID-19 pandemic crisis, there were rapid changes in government and health policies, such as the declaration of restricted zones, the control of medical equipment suppliers and COVID vaccine distribution. These changes had the potential to interfere with organizational resources. Additionally, guidelines for care and new triage screening protocols were implemented in the emergency department during the pandemic [17], leading to rapid changes in practice. In this context, clear communication with prehospital staffs prior to their arrival and the establishment of a standardized transport plan for both high- and low-risk patients became crucial [17]. However, the alterations in care provision and the boarder pandemic context might have considerably influenced ED nurses, eliciting an array of emotions such as anxiety and fear. Therefore, this study aimed to examine the effect of emotional intelligence, self-regulation, organizational resources, job demand, and job burnout on turnover intention among ED nurses during the COVID-19 pandemic crisis in Thailand.

## Methods

### Research design

This study used a cross-sectional design to investigate ED nurses’ turnover intention. Data were collected from September 1 to October 31, 2021, which was during the peak of the COVID-19 pandemic in Thailand. During this period, Thailand declared a national lockdown.

### Participants and data collection

The required sample size for this study was calculated using G power software, which showed that 322 nurses were needed to achieve 80% power, with an α-value of 0.05, and an anticipated effect size of 0.05 with nine predictors [18]. A convenient sampling method was used to select eligible registered nurses who: (1) had work experience in the ED, emergency medical services, or dispatch center for ≥ 1 year; and (2) had worked in the ED during the fourth wave of the COVID-19 pandemic in Thailand. After obtaining research approval from the relevant ethics committee, we disseminated an invitation link to the online questionnaire through the ED nurse group LINE application and Facebook pages. The invitation to complete the survey set out the objectives of the study and included a self-assessment eligibility check that participants completed before proceeding with the questions, until a projected sample size was reached based on estimates. Those who were interested in participating in the survey were asked to provide online informed consent, and then proceed to complete the questionnaire via an online platform (Google Forms). Participants were divided into two groups: (1) ED nurses worked in a controlled area and number of patients with COVID-19 visited in ED less than 20 cases a day (non-red zone group) and (2) ED nurses worked in maximum as well as strict controlled area following the government policy and number of patients with COVID-19 visited in ED more than 20 cases a day (dark-red zone group).

### Questionnaire design

#### Translation process

The Burnout Assessment Tool (BAT-12), Turnover Intention Scale (TIS-6 version 4), and Emotional Intelligence Assessment Tool were translated from their original English versions into Thai, with permission granted by the original authors. Although, BAT-12, TIS-6 and Emotional Intelligence Assessment Tool had been adapted for several countries, a translation used in Thailand had not been reported during period of the study. The original authors granted their permission via email for the translation of the tool and the use of all scales prior to the commencement of the process. Four bilingual translators were selected, including two native Thai speakers with specialized backgrounds in mental health, psychology, and occupational health nursing. These translators independently performed the forward translation of the original BAT-12, TIS-6, and Emotional Intelligence Assessment Tool from English into Thai, focusing on enhancing conceptual, semantic, and content equivalence.

During the forward translation process, the two translated Thai versions were carefully reviewed to identify any differences or discrepancies. Through open discussions and consensus, any issues or challenges encountered during translation were resolved, ensuring the accuracy and consistency of the Thai versions. Subsequently, the BAT-12, TIS-6, and Emotional Intelligence Assessment Tool in Thai were back-translated by two translators who were not exposed to the original English versions. The back-translated versions were then compared with the original English versions to evaluate any discrepancies and ensure that the intended meaning was retained in the translation.

To further establish content validity, the pre-final versions of BAT-12, TIS-6, and the Emotional Intelligence Assessment Tool (Thai) underwent a thorough review at both the item level (I-CVI) and scale level (S-CVI/Ave). Five experts in nursing administration, occupational health and psychological nursing were involved in this review process, ensuring that the translated instruments remained conceptually relevant and suitable for the target Thai-speaking population. Finally, the reliability of the translated instruments (BAT-12, TIS-6, and Emotional Intelligence Assessment Tool) was assessed through a pilot study involving 30 participants who shared comparable characteristics with the intended study population. The reliability testing aimed to determine the consistency and stability of the translated instruments in the Thai language.

#### Burnout assessment tool (BAT-12)

Burnout Assessment Tool with 23 items is a self-report questionnaire to measure burnout which was developed by Schaufeli et al. (2020) [18]. A shorter version of the Burnout Assessment Tool consists of 12 items [18], which has four dimensions: exhaustion, mental distance, cognitive impairment, and emotional impairment, are combined into a single burnout score. The internal consistency (coefficient α) of original BAT-12 score was 0.94 [19]. The psychometric properties of the BAT-12 are similar with the full version (23 items). The BAT-12 had shown the validity evidence in several counties (Italian, α = 0.87; Romania, α = 0.86 and The Polish version, α = 0.92) and have been validated during COVID-19 pandemic [20–22]. The scale consists of a 5-point Likert scale from 1 (strongly disagree) to 5 (strongly agree). A higher score on each dimension indicates a higher level of burnout. The I-CVI value of this study range from 0.80 to 1 and S-CVI/Ave value was 0.92. Cronbach’s alpha was 0.92 in this study.

#### Turnover intention scale (TIS-6 version 4)

The Turnover Intention Scale (TIS) is a questionnaire consisting of fifteen items, originally developed by Roodt (2004) based on the Theory of Planned Behavior [23]. However, a shortened version, TIS-6 was adapted from the original TIS-15 by Bothma and Roodt (2013) [23]. TIS-6 comprises six questionnaire items that participants answer a 5-point Likert scale, ranging from 1 (strongly disagree) to 5 (strongly agree) [23]. The six TIS-6 items aim to assess participants’ intention regarding turnover in their organization. The minimum possible score on TIS-6 is 6, while the maximum score is 30. A total score below 18 indicates a desire to stay in the organization, while scores above 18 indicates a desire to leave. The original TIS-6 demonstrated good internal consistency, with a Cronbach’s alpha of 0.82. Moreover, the scale has been translated into several languages, including a Chinese with an alpha value of 0.79 and a Qatar version with an alpha value of 0.80 [24, 25]. The I-CVI value S-CVI/Ave value of this study were 1.00 in this study. Cronbach’s alpha for the TIS-6 in this study were 0.90.

#### Emotional intelligence assessment tool

The original emotional intelligence (15 items) was developed by Iqbal et al. (2021) and the score covered four dimensions: self-awareness, self-regulation, work motivation, and social skills [26–28]. The Cronbach’s alpha was test in each dimension including, self-awareness (0.706), self-regulation (0.703), work motivation (0.720), and social skills (0.706) respectively [26]. In this study, the 15 questionnaire items are answered on a 5-point Likert scale from 1 (strongly disagree) to 7 (strongly agree). The scores range from 15 to 75; scores of 15–25 indicate low emotional intelligence, 26–70 indicate medium emotional intelligence, and 71–105 indicate high emotional intelligence. An example item is “I believe that emotion play an important role in everyday life” and “during COVID-19 pandemic I find it easy to share my feelings with others”. The I-CVI value of this study range from 0.80 to 1 and S-CVI/Ave value was 0.9. Cronbach’s alpha for the emotional intelligence in this study were 0.80.

#### Organizational resources and maladaptive self-regulation scale

The organizational resources and maladaptive self-regulation scale were developed by the PI based on an extensive literature review [4, 5, 7, 10, 29, 30]. Initially, we established the definition and purposes of these scales using relevant studies from the past. Subsequently, a 5-point Likert scale format was adopted for the response scale. Next, item generation was undertaken to comprehensively encompass the concepts. This included two parts: organizational resources (four items) and maladaptive regulation (four items) under the domain of organizational resources and self-regulation. In the following step, a meticulous review of both the item level (I-CVI) and scale level (S-CVI/Ave) content validity was conducted by five experts in nursing administration and occupational health. All items were refined based on the expert feedback. Finally, a reliability test was administered to 30 participants who shared similar characteristics with the intended study population.

The organizational resources section, ED nurses were asked to rate their agreement with each item using a 5-point Likert scale from 1 (strongly disagree) to 5 (strongly agree). For the organizational resources section, the scores range from 4 to 20; a higher score indicates greater organizational resources. An example item is “My hospital administrators provided enough PPE for healthcare providers.” The CVI and Cronbach’s alpha for this part of the questionnaire were 0.87 and 0.82, respectively.

The items evaluating maladaptive regulation covered psychological distress and self-undermining with scores ranging from 4 to 25. A higher score indicates greater maladaptive regulation. An example item is “I fear to take care of patients with COVID-19 infection.” The CVI and Cronbach’s alpha for these items were 0.87 and 0.82, respectively. Construct validity was not assessed for these scales, which could be considered a limitation in our measurement approach.

### Statistical analysis

The collected data were analyzed using SPSS version 22. The demographic and major variables in this study were analyzed using descriptive statistics, including percentages, means, and standard deviations. linear regression (forward stepwise method) was used to identify which variables could explain turnover intention among ED nurses during the COVID-19 pandemic, after statistical testing F - test ANOVA (linearity), Kolmogorov - Smirnov Test (normality), Box’s Test of Equality (homoscedasticity), and VIF value ≤ 3 (multicollinearity). The level of significance for this study was set at p < .05. The significant variables were entered into forward stepwise method analysis including age, self-regulation, motivation, social skills, organization resources, maladaptive regulation, and burnout (exhaustion, mental distance, cognitive impairment, emotional impairment), then started removing the least significant model caused lowest drop R^2^.

### Ethical considerations

This study received ethical approval from Chulabhorn Research Institute Ethic Committee (No.:136/2564). The objectives of the study were clearly stated for all participants. All participants provided written electronic consent before responding to the survey.

## Results

In total, 332 participants completed the questionnaire. As shown in Table 1, participants comprised two groups: the non-red zone group (n = 208) and the dark-red zone group (n = 114). Most participants in both groups were female (68% vs. 78.1%). The mean age in the non-red zone group was 34.20 years and that in the dark-red zone group was 35.64 years. Most participants were trained ED nurses or ED nurse practitioners (44.7% and 52.6% in the non-red zone and dark-red zone groups, respectively) and were not married (65.9% and 63.2%, respectively). Most ED nurses (83/114, 72.8%) in the dark-red zone group desired to leave their organization, whereas 44.7% of ED nurses (93/208) in the non-red zone desired to stay (Table 1).

**Table 1:** Characteristics of participating emergency nurses

Characteristics		Non-red zone group (n = 208)	Dark-red zone group (n = 114)
		n (%)	n (%)
**Sex**			
Male		79 (38.0)	25 (21.9)
Female		129 (62.0)	89 (78.1)
**Age, years** (mean 34.54 ± 8.78)			
**Education level**			
Bachelor’s degree		193 (92.8)	102 (89.5)
Higher than bachelor’s degree		15 (7.2)	12 (10.5)
**Training in nursing specialty program**			
No		88 (42.3)	47 (41.2)
Emergency nurse/emergency nurse practitioners		93 (44.7)	60 (52.6)
Another program		27 (13.0)	7 (6.1)
**Marital status**			
Not married		137 (65.9)	72 (63.2)
Married Divorced/widowed		63 (30.3) 8 (3.8)	41 (36.0) 1 (0.9)
**Had children**			
Yes		68 (32.7)	31 (27.2)
No		140 (67.3)	83 (72.8)
**Workplace setting**			
Secondary hospital		154 (74.0)	49 (43.0)
Tertiary hospital		53 (25.5)	61 (53.5)
University hospital		1 (0.5)	4 (3.5)
Government hospital		185 (88.9)	109 (95.6)
Private hospital		23 (11.1)	5 (4.4)
**Working hours/week**			
40		34 (15.8)	5 (4.4)
41–60		93 (43.3)	44 (38.6)
> 60		88 (40.9)	65 (57.0)
**Intention to leave their organization**		115 (55.3)	(83) 72.8
**Number of patients with COVID-19 visiting**	Median = 4, IQR = 6		Median = 20, IQR = 10

IQR = Interquartile range

Fewer patients with Covid-19 were observed in the non-red zone group compared to the dark-red zone group (Median; IQR = 4;6 vs. 20;10). The average score for turnover intention in the non-red zone group was 17.27 ± 5.81 and in the dark-red zone group was 18.39 ± 4.69. The mean emotional intelligence score was higher in the non-red zone group than the dark-red zone group (59.14 vs. 55.77). The dark-red zone group had a higher mean burnout score than the non-red zone group (35.16 vs. 32.26), but the two groups had similar mean maladaptive regulation scores (11.75 vs. 11.12) (Table 2). The Pearson’s correlation analysis showed there was a significant positive correlation between overall burnout score and turnover intention score in both groups (non-red zone group: r = .688, p < .001 vs. dark-red zone group: r = .32, p < .001). Turnover intention in the dark-red zone group was significantly negatively correlated with motivation (r = − .525, p < .001) and organization resources (r = − .157, p = .047). Turnover intention in the non-red zone group was significantly negatively correlated with age (r = − .279, p < .001), self-awareness (r = − .222, p = .001), self-regulation (r = − .242, p < .001), motivation (r = − .245, p < .001), social skills (r = − .258, p < .001), and organization resources (r = − .336, p < .001). There was also a correlation between a high maladaptive score and turnover intention (Table 2).

**Table 2:** Correlations between key study variables and turnover intention (N = 332)

Variable 1	Non-red zone group (n = 208)		Test statistic	*p*-value	Dark-red zone group (n = 114)		Test statistic	*p*-value
	Mean	SD			Mean	SD		
**Turnover intention**	17.27	5.81			18.39	4.69		
**Age, years**	34.20	9.29	−0.279	< 0.001^***^	35.64	7.59	−0.120	0.102
**Emergency experience (years)** ^**a**^	8.68	7.16	0.075	0.142	11.82	7.59	−0.705	0.293
**Emotional intelligence** Self-awareness Self-regulation Motivation Social skills	59.14 16.45 12.10 13.92 15.61	5.92 1.89 1.65 1.60 2.01	−0.166 −0.222 −0.242 −0.245 −0.258	0.008 0.001 < 0.001^***^ < 0.001^***^ < 0.001^***^	55.77 15.27 10.61 12.97 15.35	7.06 2.49 1.75 1.59 2.29	0.118 −0.019 −0.147 −0.525 −0.093	0.106 0.420 0.060 < 0.001^***^ 0.164
**Organization resources**	12.87	3.21	−0.336	< 0.001^***^	13.76	2.81	−0.157	0.047
**Maladaptive regulation**	11.12	2.53	0.282	< 0.001^***^	11.75	2.29	0.192	0.021
**Burnout**	32.26	8.94	0.688	< 0.001^***^	35.16	8.57	0.632	< 0.001^***^
Exhaustion	9.63	2.82	0.752	< 0.001^***^	10.06	2.46	0.649	< 0.001^***^
Mental distance	6.72	2.71	0.529	< 0.001^***^	9.70	2.69	0.543	< 0.001^***^
Cognitive impairment	9.22	2.45	0.555	< 0.001^***^	7.78	2.85	0.563	< 0.001^***^
Emotional impairment	6.59	2.70	0.435	< 0.001^***^	7.61	2.66	0.460	< 0.001^***^
**Number of patients with COVID-19 visiting**	3.51	2.68	0.075	0.142	20.96	7.62	0.082	0.071

Pearson’s correlation was used for the analyses. SD = standard deviation. *p < .05, **p < .01, ***p < .001.

The variables that were significantly correlated with turnover intention were entered into a linear regression (forward stepwise method). Organization resources (*β*=−0.318; p < .001; 95% confidence interval [CI]: −0.733 to − 0.416), maladaptive regulation (*β* = 0.204; p < .001; 95%CI: 0.272 to 0.662), exhaustion (*β* = 0.494; p < .001; 95%CI: 0.813 to 1.215), and cognitive impairment (*β* = 0.279; p < .001 95%CI: 0.349 to 0.820) predicted turnover intention among nurses who worked in non-red zone areas. Exhaustion (*β* = 3.254; p = .002; 95%CI: 0.182 to − 0.751) and cognitive impairment (*β* = 0.387; p < .001; 95%CI: 0.367 to 0.905) predicted turnover intention among nurses who worked in dark-red zone areas (Table 3).

**Table 3:** The determinants of organization resource, motivation, maladaptive regulation, exhaustion turnover and cognitive impairment on intention among ED nurses

Variables	B	SE	β	*T*	*p*-value	95% CI
Emergency nurses in non-red zone (n = 208) ^a^						
Organization resources Maladaptive regulation Exhaustion	−0.575 0.467 1.014	0.080 0.099 0.102	−0.318 0.204 0.494	−7.152 4.717 9.957	< 0.001^***^ < 0.001^***^ < 0.001^***^	−0.733 to − 0.416 0.272 to 0.662 0.813 to 1.215
Cognitive impairment	0.598	0.103	0.279	5.782	< 0.001^***^	0.349 to 0.802
**Emergency nurses in dark-red zone (n = 114)** ^**b**^						
Motivation Exhaustion Cognitive impairment	−1.064 0.467 0.636	0.213 0.143 0.136	−0.361 0.245 0.387	−5.000 3.254 4.686	< 0.001^***^ 0.002 < 0.001^***^	−1.486 to − 0.525 0.182 to 0.751 0.367 to 0.905

CI, confidence interval; SE, standard error; β, standardized regression coefficient. Stepwise multiple regression was used for the analysis, *p* < .05, **p < .01, ***p < .001.

^a^ R = .752, R^2^ = 0.691, adjusted R^2^ = 0.684.

^b^ R = .742, R^2^ = 0.550, adjusted R^2^ = 0.538.

## Discussion

During COVID-19 pandemic, ED nurses in the dark-red zone areas had exhaustion and cognitive impairment that positively affected turnover intention, while motivation had a negative effect on turnover intention. In contrast, organization resources had a negative effect on turnover intention, while maladaptive regulation, exhaustion, and cognitive impairment had positive effects on turnover intention among ED nurses in the non-red zone areas. During COVID-19 pandemic, most ED nurses in the dark-red zone areas and the majority in non-red zone areas desired to leave their organization, with mean scores above the midpoint (mean scores: 18.39 and 17.27, respectively). Particularly, in the dark-red zone areas, ED nurses exhibited a greater inclination to depart from their organization, in contrast to findings from a prior study conducted in China [8]. The turnover intention was reported to be 72.8% in the dark-red zone areas and 55.3% in the non-red zone areas in this study. In contrast, the previous study from China reported a lower turnover intention rate of 40.6% among emergency nurses [8]. Nurses who work in an emergency have a higher turnover intention compared to nurses in other departments. Moreover, the intention to turnover among nurses was higher when compared with before COVID-19 pandemic [25]. This higher turnover intention can be attributed to factors related to their unique working environment, such as dealing with critical patients and encountering challenges with low understanding [8, 16]. ED nurses were directly involved in the management of the COVID-19 pandemic, their pandemic-related job demand and fear of COVID-19 were higher than nurses in other departments [16, 31].

Moreover, previous studies reported that burnout was a strong predictor of turnover intention among frontline nurses during the COVID-19 pandemic [7, 9, 25]. Our findings support the notion that burnout, specifically feelings of exhaustion and cognitive impairment, play a significant role in ED nurses’ intention to leave their organizations. Our results also showed ED nurses in non-red zone areas during COVID-19 pandemic had low organization resources and high maladaptive regulation, which increased their intention to leave their organization. This result was consistent with previous research that suggested uncertainty of organization resources may promote exhaustion and maladaptive regulation among employees in response to their job strain [4, 6, 32]. During the COVID-19 pandemic in Thailand, vaccines, other materials, and PPE were contributed by the government with priority given to healthcare workers in high-risk areas. Therefore, the lack of COVID-19 vaccines and PPE in non-red zone areas might have promoted unsafe workplaces that encouraged individual maladaptive regulation. The high level of maladaptive regulation among ED nurses in non-red zone areas could be explained by the low daily numbers of COVID-19 cases reported in these areas, as ED nurses might have been unfamiliar with care delivery or management for COVID-19 patients because the process of care was not routine when compared with ED nurses in dark-red zone areas. Therefore, hospitals should focus on good preparedness policies, including sufficient medical equipment, PPE stockpiles, and ongoing education and refresher courses on transmission and prevention of communicable diseases in the ED to keep up these important skills [6, 28].

Our finding demonstrated that the number of patients with COVID-19 increased rapidly after the onset of the COVID-19 pandemic in dark-red zone areas compared with non-red zone areas. Although our findings showed the number of COVID-19 patients visiting the ED was not related to turnover intention, the large volume of these patients at the peak of pandemic, limited space, limited resources, and ED staff limitations might have impaired nurses’ ability to fully focus on patient care tasks. ED overcrowding was complicated by serious problems finding beds in intensive care units or in cohort wards for patients who needed to be hospitalized, which further obstructed patient flow through the ED [11]. In addition, issues related to the COVID-19 pandemic occurred at hospitals in the capital city, Bangkok, as well as in the surrounding provinces. There were many procedures to reduce the spread of COVID-19, although the number of negative pressure rooms was limited in dark-red zone areas. PPE was the main measure to safeguard against virus infection, but long hours wearing PPE was associated with discomfort [30], especially as wearing PPE in a hot environment may increase the risk for heat stress. In addition to increasing job demand, the pandemic resulted in ED nurses’ exposure to multiple factors stimuli, especially a stressful workplace environment [31, 33–35]. Therefore, the compounding factors that affected ED nurses in dark-red zone areas during the COVID-19 pandemic might have promoted high levels of emotional exhaustion. However, good emotional intelligence may prevent negative effects of psychological risk factors, especially burnout [3, 36, 37]. Our result show that ED nurses having a high level of emotional intelligence, low work motivation can still contribute to an increased rate of turnover intention. In other words, even if nurses possess the necessary emotional intelligence skills, if they lack motivation in their work, they may be more likely to consider leaving their positions. Motivated nurses who feel empowered and supported by their organization are more likely to be engaged and committed to their work. They are driven to provide high-quality care and achieve positive patient outcomes [16].

Furthermore, this study show that cognitive impairment significantly influenced intention to turnover among ED nurses during COVID-19 pandemic in both dark red zone and non-red zone areas. A major contributor to this may be ED overcrowding, which is a complex situation that remains unresolved in Thailand [38]. However, ED nurses were exposed long-term overcrowding, high levels of work stress, burnout, and unsafe workplace environments before the onset of the COVID-19 pandemic [8, 11, 38]. During the pandemic, the combination of workload, fear of COVID-19, exhaustion, and emotion strain might have increased cognitive impairment among ED nurses. Previous studies noted that to reduce the risk for cognitive impairment, healthcare organizations should promote workplace safety in the ED and a work environment that allows ED nurses to reinforce their resilience, which may be a protective factor against adverse workplace conditions [31, 33, 39].

This study had several limitations. Firstly, we used a cross-sectional design during COVID-19 pandemic that provided a snapshot of one time point only and did not enable us to determine causality. The main result of this study showed that exhaustion and cognitive impairment predicted intention to leave their organization among ED nurses. Unfortunately, few studies investigated exhaustion, cognitive impairment, and turnover intention among ED nurses in Thailand before the onset of the COVID-19 pandemic, meaning a pre-/post-pandemic comparison was not possible. Secondly, national policies regarding public health and organization resources were dynamic during the study period, which could have impacted turnover intention among ED nurses. Thirdly, the effect size observed in this study may be of limited magnitude, potentially impeding the practical significance of the identified relationship. Therefore, further studies are needed to explore the long-term impacts of the pandemic on ED nurses. Finally, generalization of the findings is limited because a non-probability sampling is a bias sampling method and sample included a limited number of ED nurses working in private hospitals. Further studies are needed, including a qualitative investigation, to explore employee career advancement and cognitive impairment among ED nurses during the pandemic period.

The findings of this study offer input on how to support ED nurses during a pandemic or similar crisis to reduce their turnover intention. The diversities and complex conditions during the COVID-19 pandemic were challenges for nurse managers and nurses who worked in EDs. Therefore, healthcare organizations, nurse administrators, and ED nurse managers could provide interventions that reduce exhaustion among nurses and address problems in dealing with work-related stress. Providing training in regard to care and management of transmissible diseases, giving up-to-date health policy, and redesign ED services for crisis situations may help ED nurses to cope with complex and unpredictable situations in the ED context.

## Conclusion

The factors that predicted turnover intention differed between ED nurses during COVID-19 pandemic. Exhaustion and cognitive impairment positively affected turnover intention while motivation negatively affected turnover intention among ED nurses in dark-red zone areas. Among ED nurses in non-red zone areas, organization resources had a negative effect on turnover intention and maladaptive regulation, while exhaustion, and cognitive impairment had positive effects on turnover intention. Hence, its varying influence on ED nurses across different zones underscores the significance. Gaining insights into the determinants of turnover intention among ED nurses can inform strategic workforce planning over the long term. Policymakers can optimize resource allocation by concentrating on regions with the greatest turnover intention. This comprehension empowers them to establish an environment that nurtures, retains, and recognizes the contributions of ED nurses during emergencies and crises, thereby enhancing the overall well-being of these health care professionals.

## Data Availability

Study data and questionnaires are available from the corresponding author upon reasonable request.

## Abbreviations

ED: emergency department
PPE: personal protective equipment
